# Supplementary material for: Up to a 15-Year Survival Rate and Marginal Bone Resorption of 1780 Implants with or without Microthreads: A Multi Center Retrospective Study
Source: J Clin Med. 2023 Mar 21;12(6):2425. doi: 10.3390/jcm12062425 (PMC10057595; doi:10.3390/jcm12062425)
Supplement: Supplementary file 1 [file jcm-12-02425-s001.zip › jcm-2287475-supplementary.pdf]

Supplementary Material

Table 1. The results of linear mixed model at 6 year follow up

		Univariable model	
		BETA (SE)	*p*-value
Implant	IT	Ref	
	IU	0.015(0.008)	0.06
Gender	Male	Ref	
	Female	0.009(0.006)	0.18
Diameter	< 4 mm	Ref	
	4–5 mm	−0.011(0.016)	0.47
	> 5 mm	−0.027(0.017)	0.12
Length	< 8.5 mm	Ref	
	8.5~10 mm	−0.021(0.023)	0.34
	> 10 mm	−0.02(0.023)	0.38
Prosthesis type	Single	Ref	
	Consecutive	0.007(0.01)	0.47
	Bridge	0.001(0.009)	0.91

Ref: reference
